# Near-Field Topology-Optimized Superchiral Metasurfaces for Enhanced Chiral Sensing

**DOI:** 10.1021/acs.nanolett.5c05820

**Published:** 2026-01-16

**Authors:** Zhongjun Jiang, Soyaib H. Sohag, You Zhou

**Affiliations:** Department of Physics and Optical Science, University of North Carolina at Charlotte, Charlotte, North Carolina 28223, United States

**Keywords:** chiral sensing, superchiral field, metasurfaces, topology optimization, circular dichroism

## Abstract

The detection and discrimination of molecular chirality are essential for the advancement of pharmaceutical and biological applications. While nanophotonic platforms offer a route to enhance chiral light–matter interactions, existing device concepts for chiral sensing remain heuristic, resulting in limited chiral enhancement and control over chiral hotspot placement within nanostructures. Here, we introduce an inverse design framework that directly optimizes superchiral near fields in photonic nanostructures and demonstrate its powerful opportunities for enantioselective analysis. We first show that freeform achiral metasurfaces can be optimized to achieve an 820-fold chiral density enhancement with fully customizable chiral hotspot placement for direct molecular interaction. We then leverage this platform to demonstrate ultrasensitive detection of chiral analytes and quantitative readout of the chiral concentration and enantiomeric excess in chiral mixtures. Our framework offers a generic route to realizing superchiral nanophotonic platforms compatible with diverse spin-based photonic materials for valleytronics, chiral emission control, and topological photonics.

## Graphical abstract

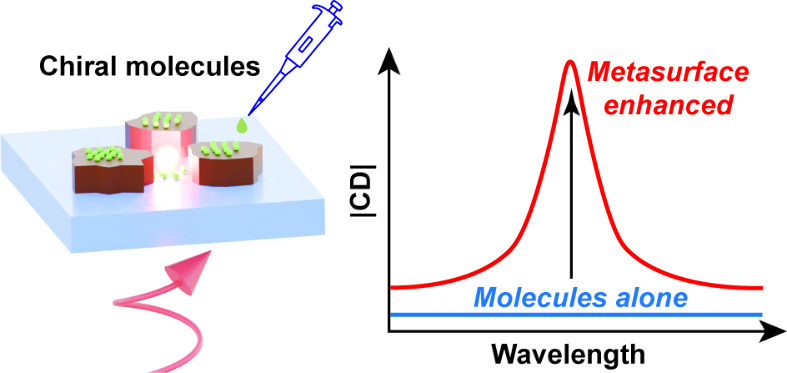

Chirality is a geometric property of an object that cannot be superimposed on its mirror image. Such spatial asymmetry at the molecular scale gives rise to enantiomeric pairs that often exhibit distinct biological and pharmacological properties.^1^ For chiral analytes, the enantiomeric composition is typically identified by circular dichroism (CD),^2,3^ based on the differential absorption of right- and left-circularly polarized light (RCP and LCP). However, the enantioselective sensitivity of conventional CD spectroscopy is inherently weak due to the large mismatch between the molecular dimensions and the helical pitch of circularly polarized light (CPL). Rapid advances in nanotechnology have enabled nanophotonic platforms to resonantly enhance light–matter interactions,^4–6^ offering a route to enhanced enantioselective detection in a compact manner.^7–12^ These developments have led to various nanophotonic concepts for applications spanning from far-field wavefront shaping^13–19^ to functionalities enabled by enhanced optical near fields.^20–24^ More recently, nanophotonic technologies have been increasingly applied in emerging areas such as sensing,^25–27^ biomedical imaging,^27^ analog computing,^13,28–30^ and additive manufacturing.^31,32^ However, a design framework that effectively enhances optical chirality in photonic nanostructures remains elusive.

Achieving sensitive enantioselective detection requires photonic sensors that confine the helical twist of CPL to subwavelength dimensions, thereby generating superchiral near fields.^33–36^ The optical chiral density is defined as^9,37^

1 C=−k02c0Im(E·H*)

where **E** and **H** are the complex electric and magnetic field vectors, and *k*_0_ and *c*_0_ are the wavenumber and speed of light in free space, respectively. As made explicit by eq 1, achieving large superchiral fields requires the excitation of strong, localized **E** and **H** fields that are (i) spatially and spectrally overlapped, (ii) directionally aligned, and (iii) phase-shifted by π/2. To facilitate direct interaction with target analytes, the chiral hotspots should also form in the open regions of the nanostructures, where the molecules reside. Furthermore, in contrast to chiral metasurface concepts that aim to enhance far-field structural CD,^38–42^ a chiral sensor should exhibit minimal chiroptical background to isolate the intrinsic analyte response,^8,37,43,44^ which necessitates geometrically achiral layouts with mirror or *C*_*n*_ (*n* > 2) rotational symmetry. Achieving these criteria through nanostructure engineering is a highly nontrivial task due to the lack of precise analytical correlations between nanoscale geometries and near-field distributions. To date, the existing chiral sensing paradigm has relied on an empirical identification of simple and physically intuitive geometries described by a small set of parameters, followed by fine-tuning these parameters through full-wave simulations to reach a local optimum in a highly constrained design space.^37,43–47^ While this approach has produced an alphabet of meta-atom templates that form the foundation of chiral sensing research, it offers limited control over both the magnitude and the spatial placement of chiral hotspots. In addition, these strategies explore only a small subset of the vast freeform design space accessible to nanophotonic structures, which is often insufficient to realize complex, multifunctional chiral responses. As a result, the chiral enhancements in these rationally designed devices remain modest and are predominantly confined within the nanostructures.^37,43–52^

Here, we develop a novel inverse-design framework that explicitly engineers superchiral near fields in dielectric metasurfaces. While inverse design techniques have been widely used to shape the far-field responses of metasurfaces,^53–60^ our approach bridges intricate near-field engineering with freeform topology optimization by targeting near-field chiral figures-of-merit (FoM). The schematic workflow of our design concept is shown in Figure 1. First, the spatial profile of the superchiral field is nucleated by defining the FoM as Im­(**E**·H*) at a probe point inside the metasurface (Figure 1 left), thereby directly linking maximization of this FoM to enhanced local chiral density. We chose a pointwise FoM because it provides a numerically stable objective function for gradient-based optimization. Interestingly, we find that optimizing the chiral density at a single point alone is sufficient to induce robust, spatially extended chiral-field enhancement over the entire metasurface area. The nanostructure design space is constrained to be geometrically achiral, so the resulting far-field CD arises solely from the chiral analyte. The frequency, handedness, and spatial position of the FoM can be tailored at will, allowing chiral hotspots to be placed outside the metasurface structures for direct molecular interaction (Figure 1, middle). To couple free-space waves to the desired chiral modes, we develop a near-field topology-optimization framework based on the adjoint-variable method,^61,62^ in which a combination of far- and near-field excitation sources are utilized for forward and adjoint simulations.^63^ Our method explores a large freeform design space by rigorously accounting for the complex relationships between optical near fields and nanoscale geometry, thereby pushing the chiral-enhancement limits beyond empirical, template-based designs. Through multiobjective optimization, our framework naturally extends to devices hosting multifunctional chiral responses for broad spin-selective photonic applications.^64,65^ Leveraging these capabilities, we realize freeform superchiral metasurfaces capable of the ultrasensitive detection and discrimination of chiral molecules (Figure 1, right).

**Figure 1:**
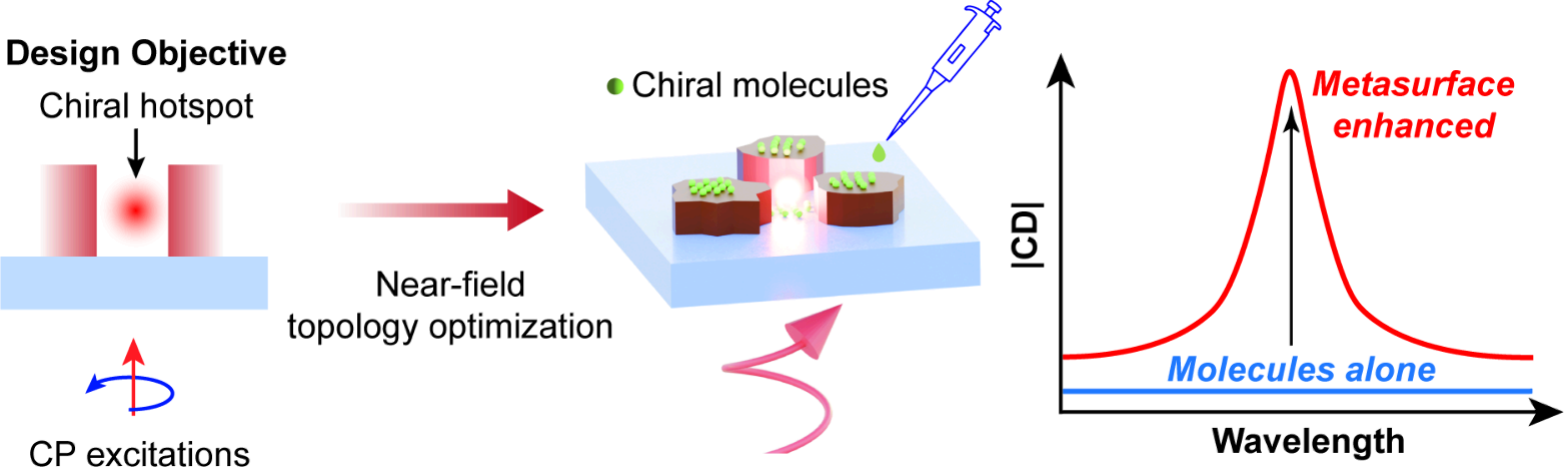
Framework for engineering superchiral near fields in freeform metasurfaces. Optical chiral density inside the photonic nanostructure is defined as the design objective and maximized under circularly polarized (CP) illumination via near-field topology optimization (left). The resulting superchiral hotspots are formed outside the nanostructures (middle), enabling ultrasensitive detection of chiral molecules (right).

The metasurface consists of a subwavelength array of free-form meta-atoms arranged on a square lattice with a period of 720 nm (Figure 2a). We choose silicon as the dielectric material due to its low loss, high refractive index, and ability to support Mie-type resonant modes.^5,66,67^ A 460 nm thick silicon layer is selected to support the excitation of multipolar resonances consisting of spectrally overlapped electric and magnetic modes. The FoM is defined as the pointwise quantity Im­(**E**·H*) that measures local chiral density and is maximized at the unit cell center under free-space CPL illumination. We enforce mirror symmetry on the freeform design space to impose geometric achirality (Figure 2b, left), thereby suppressing the structural chiroptical background. To ensure that the chiral hotspot can form outside the silicon nanostructures (Figure 2b, right), we impose a small air void centered at the FoM probe point as the molecular host region.

**Figure 2:**
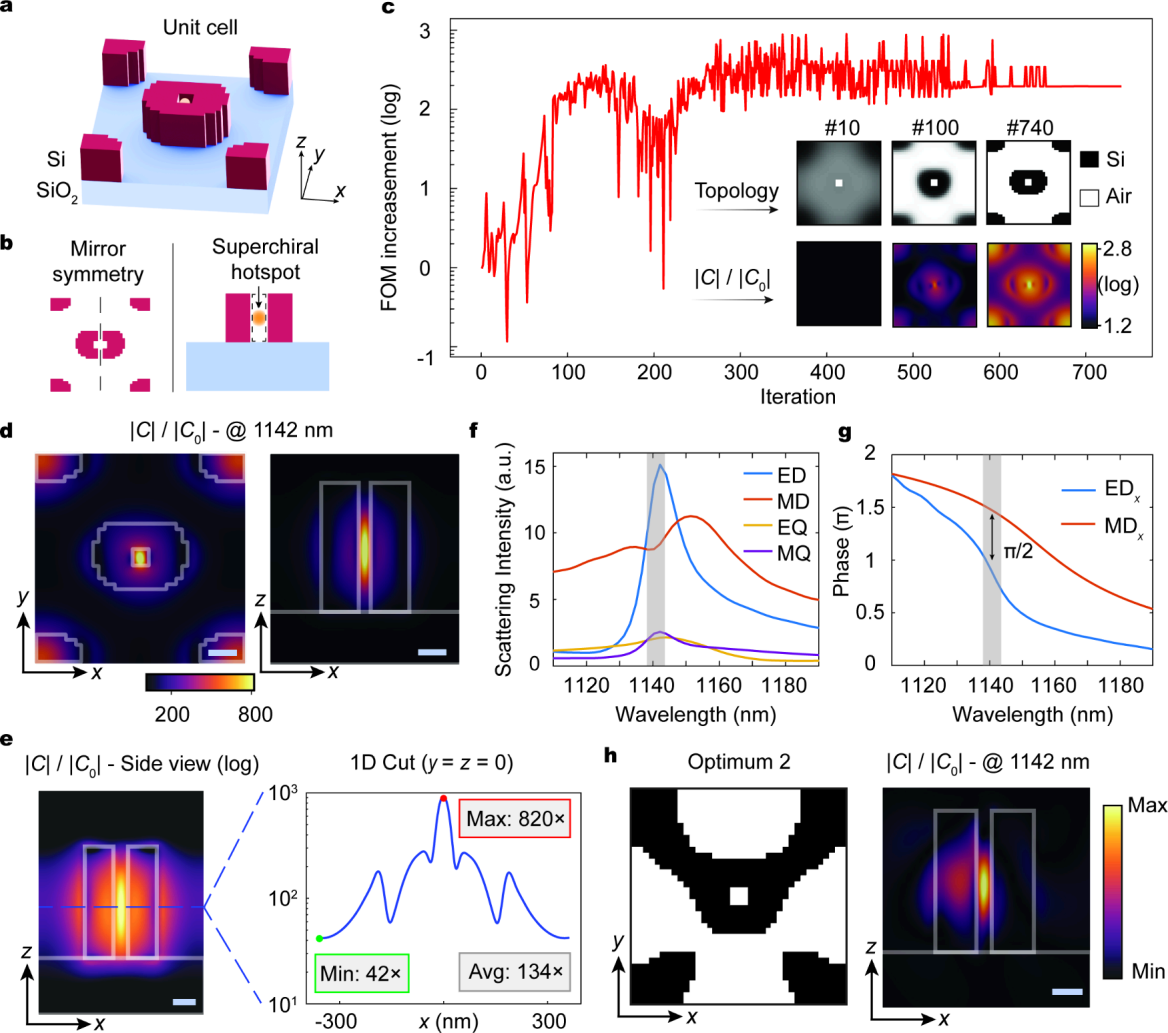
Superchiral freeform metasurfaces. (a) Unit cell of the optimized metasurface. (b) Key design considerations. First, mirror symmetry constraint is applied to the design space to ensure a geometrical achiral layout (left). Second, a small air void is imposed and centered at the target hotspot to enable direct molecular access (right). (c) Optimization trajectory showing the enhancement of the figure-of-merit (FoM) during optimization. Insets: structural and normalized chiral density (log scale) evolution within the unit cell. (d) Top view (left) and side view (right) of chiral density distributions (linear scale) overlaid on the nanostructures, confirming a strongly localized chiral hotspot confined within the air gap. (e) Log-scale side-view chiral density map and its one-dimensional horizontal cut. (f) Multipolar decomposition of the optimized metasurface. Abbreviations: ED (electric dipole), MD (magnetic dipole), EQ (electric quadrupole), MQ (magnetic quadrupole). (g) Phase delays confirming a π/2 phase difference between the ED and MD resonances. (h) A second freeform achiral design obtained from a random initialization (top view) and its normalized chiral-density distribution (side view). Scale bars: 100 nm.

We employ the adjoint solver in the open-source finite-difference time-domain package Meep,^68^ coupled with the Adam optimizer in PyTorch,^69^ to perform gradient-descent optimization (see optimization details Supporting Information section 1). The optimization trajectory showing FoM enhancement (log scale) and topology evolution is presented in Figure 2c. Over the course of optimization, the FoM increases consistently and ultimately reaches more than an 800-fold enhancement compared with the starting point. The inset shows the topology transformation that evolves from a grayscale permittivity distribution to a binary free-form structure consisting of air and silicon. The top-view chiral density maps show a steady increase over the course of iteration, ultimately leading to a strong chiral-field enhancement in the final binarized device. The top- and side-view overlays of the superchiral near field on the nanostructure (Figure 2d) further confirm a localized chiral hotspot confined within the air gap. It is important to note that the dominant hotspot confined to a small volume arises from the strong enhancement at the probe point, which visually overshadows the surrounding regions. However, the superchiral mode profile is spatially extended beyond the probe location with a strong chiral-field content visible in the log-scale representation. As shown in Figure 2e, the side-view chiral-density profile and its one-dimensional horizontal cut reveal broad spatial coverage, with a 134-fold chiral enhancement averaged over the entire unit-cell area. The local enhancements range from 42× to 820×, with even the weakest enhancement exceeding many state-of-the-art rationally designed structures.^34,44,47,49,51^ Such volumewise superchiral fields ensure a large analyte overlap and naturally accommodate diverse molecular distribution scenarios, thereby mitigating the intrinsic uncertainty in chiral molecular delivery.

To uncover the resonant mechanisms behind the enhanced chiral field, we perform multipolar decomposition of the metasurface near fields from the current density distributions induced in the nanostructures using the open-source software MENP.^70^ The multipolar decomposition as a function of wavelength (Figure 2f) reveals the excitation of two dominant dipole modes in the form of spectrally overlapped in-plane electric *E*_*x*_ and magnetic *H*_*x*_ resonances, along with additional higher-order modes that are an order of magnitude weaker and thus contribute negligibly to the overall chiral enhancement. The relative phase retardation between the two dipole modes, shown in Figure 2g, is roughly π/2 at the designed wavelength, satisfying the condition for enhanced chiral density that scales as Im­(**E**·H*) (see chiral density enhancement as a function of wavelength in Supporting Information section 2). The simultaneous realization of directional field overlap and relative phase matching highlights the capability of our topology optimization framework to naturally discover geometries that satisfy multiple stringent requirements, with all geometric elements, including both the central holey structure and the surrounding nanodisks, collectively playing a critical role (see Supporting Information section 3). It is worth noting that such a geometrically smooth structure represents only one local optimum in a nonconvex optimization landscape, which we selected to promote robust fabrication. Notably, our approach enables exploration of a substantially larger freeform achiral design space. As shown in Figure 2h, we further optimize the same chiral FoM from a randomly initialized permittivity distribution and obtain a physically nonintuitive achiral geometry exhibiting similar chiral hotspot formation (see additional freeform superchiral metasurface designs in Supporting Information section 4).

To experimentally validate the design, we fabricate the optimized metasurfaces on a 462 nm-thick amorphous-silicon film grown by plasma-enhanced chemical vapor deposition. The freeform nanostructures are defined using standard electron-beam lithography and reactive-ion etching (see fabrication details in Supporting Information section 5). Scanning electron microscopy images of the fabricated device are presented in Figure 3b and show well-defined geometric features consistent with the design. We first evaluate the far-field response of the metasurface without a chiral overlayer (Figure 3a). As shown in Figure 3c, the simulated transmittance spectra reveal two dips corresponding to excitation of electric and magnetic dipole modes shown in Figure 2f. The far-field transmittance spectra are characterized under CPL illumination from a supercontinuum laser source, with the transmitted light collected by a customized imaging system and relayed to a spectrometer (details of measurement setup in Supporting Information section 5). The measured transmittance shown in Figure 3d exhibits good agreement in the spectral line shape with the simulation.

**Figure 3:**
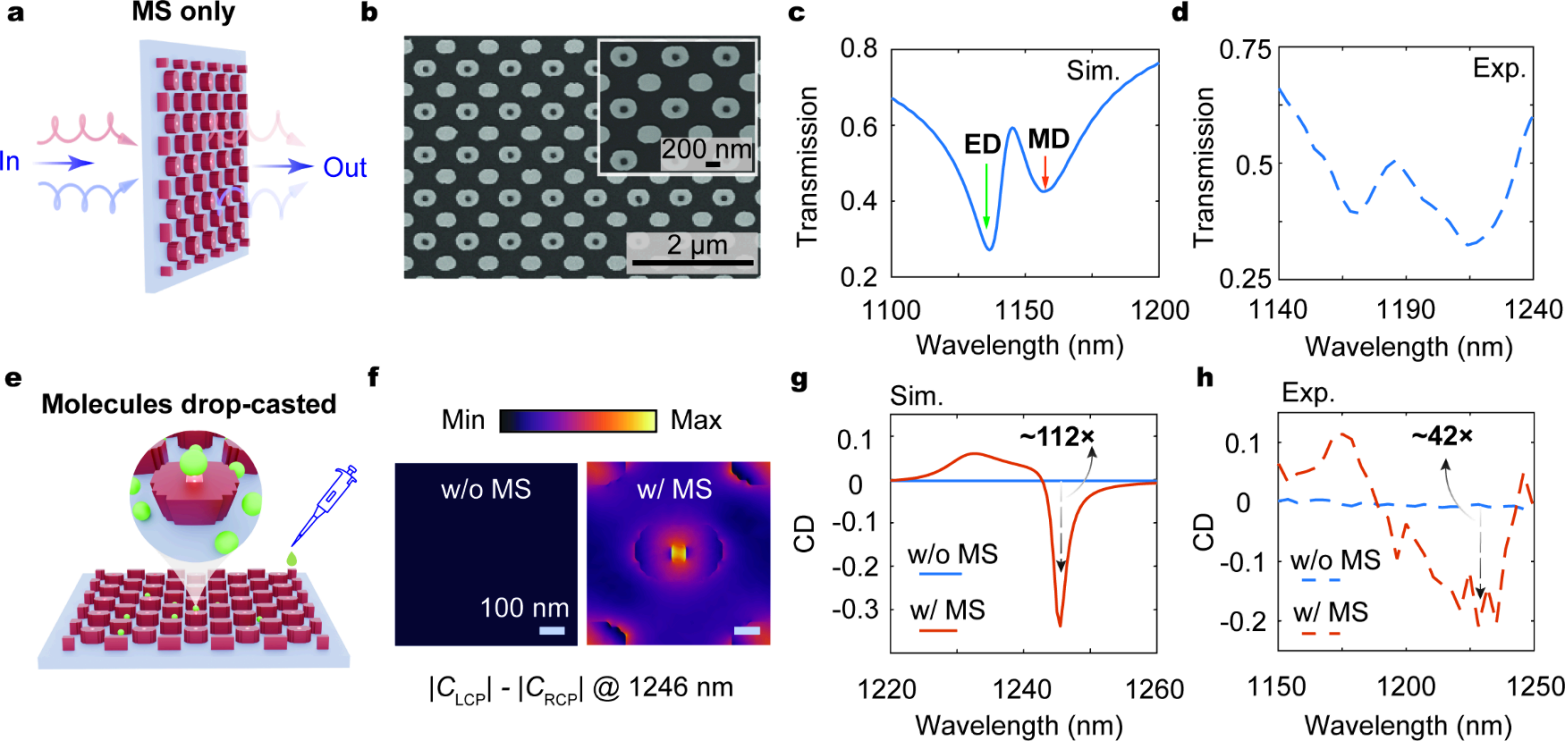
Experimental demonstration of chiral sensing. (a) Far-field device characterization. (b) Scanning electron microscope images of the fabricated metasurface sensor. Simulated (c) and measured (d) transmittance spectra of the metasurface without chiral analyte. (e) Chiral sensing by drop-casting a chiral overlayer onto the metasurface. (f) Differential chiral density distribution Δ*C* = *|C*_L_| – *|C*_R_| at 1246 nm for molecules without (left) and with (right) a metasurface. Simulated (g) and experimental (h) CD spectra for molecules on the metasurface (orange) and on a bare glass substrate (blue).

We further validate the device’s sensing performance by coating it with a thin chiral overlayer (Figure 3e). The molecules are modeled as an isotropic chiral medium with refractive indices *n* and Pasteur parameter κ. We note that the exact optical constants of dilute chiral solutions are difficult to determine precisely due to intrinsically weak chiral signals and their strong dependence on concentration and solvent environment. Thus, we select representative parameters for a weak chiral overlayer solely to probe the sensor sensitivity, specifically *n* = 1.34 – 0.001*i* and κ = (7 – 1.5*i*) × 10^–3^, uniformly coated on the metasurface. The resulting coupled electromagnetic response is obtained from full-wave simulations in COMSOL Multiphysics. We first compute the optical chiral density *C* inside the nanostructures and evaluate the LCP-RCP difference, Δ*C* = *|C*_L_| – *|C*_R_ *|*, as a measure of metasurface-enhanced chiral sensitivity. As shown in Figure 3f, the top view of the Δ*C* distribution exhibits a pronounced localized peak with a more than 10^3^-fold enhancement over the native molecular signal. We define CD as (*T*_L_ – *T*_R_)/(*T*_L_ + *T*_R_) in this work, where *T*_L,R_ denotes the transmission under LCP/RCP. The simulated CD spectra (Figure 3g) reveal a 112-fold increase in peak CD for the metasurface sensor compared to a bare substrate (see the CD spectrum of the initial design as another benchmark in Supporting Information section 6). The CD peak red-shifts from 1142 nm (bare metasurface) to 1246 nm after introducing the chiral overlayer, mainly due to the associated refractive-index perturbation. While we choose a particular Pasteur parameter here to probe the sensor sensitivity, our sensing modality is robust over a wide range of κ values (see chiral sensing performance for varying Pasteur parameter values in Supporting Information section 7).

We then experimentally assess the sensing performance by coating the metasurface with a thin layer of (*S*)-(+)-1,2-propanediol solution (see Supporting Information section 5 for analyte preparation). The measured CD spectrum (Figure 3h) shows more than 42× enhancement in CD contrast, with a line shape in good agreement with simulation. We attribute the broadened experimental spectral line width to increased absorption in the chiral solution and reduced CD enhancement to fabrication imperfections, a nonuniform chiral overlayer, and the simplified simulation model (see additional chiral sensing results for metasurfaces with geometric perturbations in Supporting Information section 8). Further improvements can be achieved by ensuring uniform analyte delivery and imposing more stringent feature-size constraints to reduce sensitivity to fabrication imperfections, for example, by co-optimizing fabrication-deviated geometries^57,71^ or using reparameterization techniques^72–76^ to impose fabrication constraints beyond density-based topology optimization. We note that while structural CD from the metasurface is, in principle, eliminated by its geometrically achiral layout residual device chirality may still arise from oblique incidence due to slight substrate tilt or other measurement asymmetries. To assess these effects, we characterize the metasurface without chiral overlayers and observe negligible CD under the same measurement conditions (Supporting Information section 9).

Lastly, we investigate the enantioselectivity of the device as a function of concentration and demonstrate its ability to resolve enantiomeric excess for arbitrary chiral mixtures. We first perform full-wave simulations of the metasurfaces covered with an enantiopure chiral overlayer for different values of the Pasteur parameter. We use the scaled Pasteur parameter κ/κ_0_ with κ_0_ = −(2.3 – 0.5*i*) × 10^–3^; thus κ/κ_0_ = +1 (−1) denotes 100% *R*- (*S*-)­enantiomer. Figure 4a shows the Δ*C* distributions inside the nanostructures for different κ/κ_0_, revealing chiral contrast that grows with the molecular concentration, consistent with the monotonic increase in the CD spectra (Figure 4b). The extracted peak CD from each concentration (Figure 4c) shows linear scaling with κ/κ_0_, providing a quantitative readout of the enantiomeric concentration.

**Figure 4:**
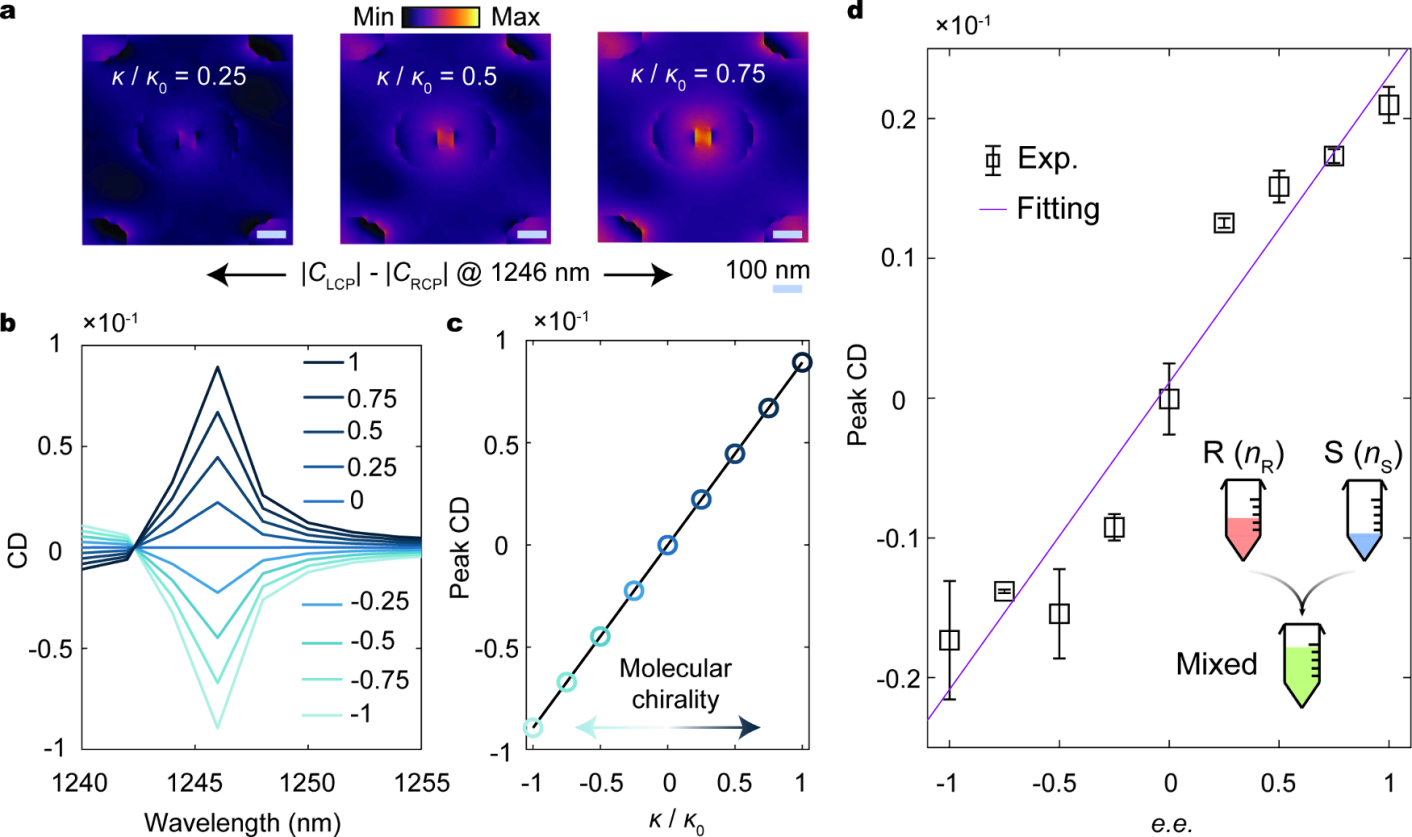
Chiral sensor for enantiomeric excess and purity readout. (a) Differential chiral density distribution Δ*C* = *|C*_L_| – *|C*_R_| at 1246 nm for molecules at varying compositions. (b) Simulated circular dichroism (CD) spectra for different values of the scaled Pasteur parameter κ/κ_0_, where κ_0_ = −(2.3 – 0.5*i*) × 10^–3^. (c) Peak CD as a function of κ/κ_0_. (d) Measured peak CD for different enantiomeric compositions of (*R*)- and (*S*)-1,2-propanediol.

We further extend our platform to determine the enantiomeric excess and purity in chiral molecular mixtures. This demonstration reflects practical pharmaceutical scenarios, where most drugs are prepared as mixtures of enantiomers, and the ability to accurately assess enantiomeric purity is crucial for drug synthesis and pharmaceutical development.^77–79^ The enantiomeric excess (*e.e*) is defined as a measure of purity:^80^

$$e.e=\frac{C_{R}\cdot V_{R}-C_{L}\cdot V_{L}}{C_{R}\cdot V_{R}+C_{L}\cdot V_{R}}$$

where *C*_R_ and *C*_L_ denote the concentrations of R- and L-handed chiral molecule solutions, respectively, and *V*_R,L_ is the volume of *R*- (*L*-)­handed molecules. We prepared mixtures of (*R*)- and (*S*)-1,2-propanediol at varying compositions while keeping the total volume fixed at 0.80 mL. The enantiomer fraction was varied over the full composition range, from a purely L-enantiomer to a purely R-enantiomer, with the complementary fraction provided by the opposite enantiomer. Each mixture was adsorbed onto the sensor to record the CD signal. Between measurements, the residual adsorbates were removed by sequentially rinsing the surface with dimethyl sulfoxide (DMSO), acetone, isopropyl alcohol (IPA), and deionized water. Repeated measurements were performed to confirm that no residual chiral molecules remained after this cleaning procedure (see testing results over multiple rinsing cycles in Supporting Information section 10). Figure 4d shows the peak CD as a function of *e.e*, revealing a linear relationship with the concentration imbalance between the two enantiomers. The *e.e* detection limit here is around 10% of the full range and is constrained by system-level experimental noise, including limited detector quantum efficiency, spectrometer sensitivity, and surface scattering from the imaging and relay optics. To better isolate the intrinsic chiral signatures, beam chopping combined with lock-in amplification can be used to enhance the signal-to-noise ratio.^47^ These results highlight the robustness of our approach for quantitative readout of the enantiomeric concentration and purity. The sensing platform can be further improved at the system level by integrating the sensor with microfluidic flow cells^81^ that allow uniform, actively controlled delivery of chiral analytes.

In summary, we introduce and demonstrate a near-field topology optimization framework for creating and tailoring superchiral near fields in free-form dielectric metasurfaces. Our approach directly optimizes local chirality density in photonic structures by rigorously accounting for the complex relationships between optical near fields and nanoscale geometry. Compared with conventional template-based designs, our framework offers two distinct advantages for ultrasensitive enantioselective analysis. First, it efficiently explores a large freeform design space that can be combined with global optimization algorithms^82,83^ to push the chiral enhancement limits. Second, it enables designer control over chiral hotspot locations within the nanostructure to facilitate direct molecular interaction. The chiral sensing performance can be further enhanced by targeting multiple chiral hotspots or maximizing the surface-averaged chiral density across the nanostructures. Moreover, multiwavelength optimization can extend the chiral response over a broader spectral window, thereby broadening the range of compatible analytes. In addition to enantioselective analysis, metasurfaces supporting enhanced chiral near fields can be integrated with a wide range of spin-based photonic materials to advance emerging areas such as valleytronics,^64,65^ chiral emission control,^84–86^ and topological photonics.^87^ Furthermore, our framework can be extended to devices hosting complex near-field modal profiles,^63,88–92^ thereby providing a generic route to resonant photonic platforms for applications spanning spontaneous-emission control,^93–96^ nonlinear optics,^22,23,97,98^ optomechanics,^99,100^ and photochemistry,^101,102^ where strong light–matter interactions are required.

## Supplementary Material



## Data Availability

The data that support the plots within this paper and other findings of this study are available from the corresponding authors upon reasonable request.

## Notes

Supplementary Sections 1–10: (1) Optimization workflow; (2) Chiral density spectrum; (3) Freeform element contribution analysis; (4) Additional freeform superchiral metasurface designs; (5) Experimental methods; (6) Chiral sensing performance of the initial design; (7) Chiral sensing with varying Pasteur parameter values; (8) Chiral enhancement under geometric perturbations; (9) Assessment of residual metasurface chirality; (10) Assessment of residual chiral molecules from the cleaning procedure (PDF)
