# Supplementary material for: Near-Field Topology-Optimized Superchiral Metasurfaces for Enhanced Chiral Sensing
Source: Nano Lett. 2026 Jan 16;26(3):1109–17. doi: 10.1021/acs.nanolett.5c05820 (PMC12856900; doi:10.1021/acs.nanolett.5c05820)
Supplement: Supplementary file 1 [file nl5c05820_si_001.pdf]

Supplementary Information for:

Near-Field Topology-Optimized Superchiral Metasurfaces for Enhanced Chiral Sensing

Zhongjun Jiang, Soyaib H Sohag, and You Zhou*

Department of Physics and Optical Science, University of North Carolina at Charlotte, Charlotte, NC, USA, 28223

*Email: yzhou33@charlotte.edu

Section 1: Device optimization workflow

The optimization workflow (Fig. S1) starts from a grayscale topology profile. We use a combination of conic spatial filtering (with a radius of $l/(2\sqrt{\eta_e - 0.5})$ determined from the minimum length scale l and the desired eroded threshold point η_e), and Heaviside projection to enforce minimum feature size constraints and to gradually binarize the topology during optimization. In this work, two additional constraints—a global mirror symmetry and a fixed air void—are applied to the design region to construct an achiral metasurface that supports superchiral hotspots outside the nanostructures. The geometry is evaluated through full-wave simulations using the finite-difference time-domain–based open-source software package Meep (v1.29.0). In this work, the figure of merit is defined as the pointwise quantity $\mathrm{Im}(\mathbf{E} \cdot \mathbf{H}^*)$ that measures local chiral density. Two simulations, involving a forward and a near-field adjoint simulation, are performed to obtain the gradient that guides the design evolution. In the forward simulation, the EM fields ($\mathbf{E}$, $\mathbf{H}$) along with the figure of merit F (here, the optical chiral density) are calculated with a far-field circular polarized excitation source. In the adjoint simulation, the adjoint fields are obtained by a similar simulation set-up but with the source replaced by near-field electric $\mathbf{P}$ ($\mathbf{P} = \partial F/\partial \mathbf{E}$) and magnetic $\mathbf{M}$ ($\mathbf{M} = -(1/\mu_0) \cdot \partial F/\partial \mathbf{H}$) dipole sources. The design topology is updated

from the gradient via the Adam optimizer (PyTorch, v2.4.0), and the iteration continues until the optimization converges or meets our design targets.

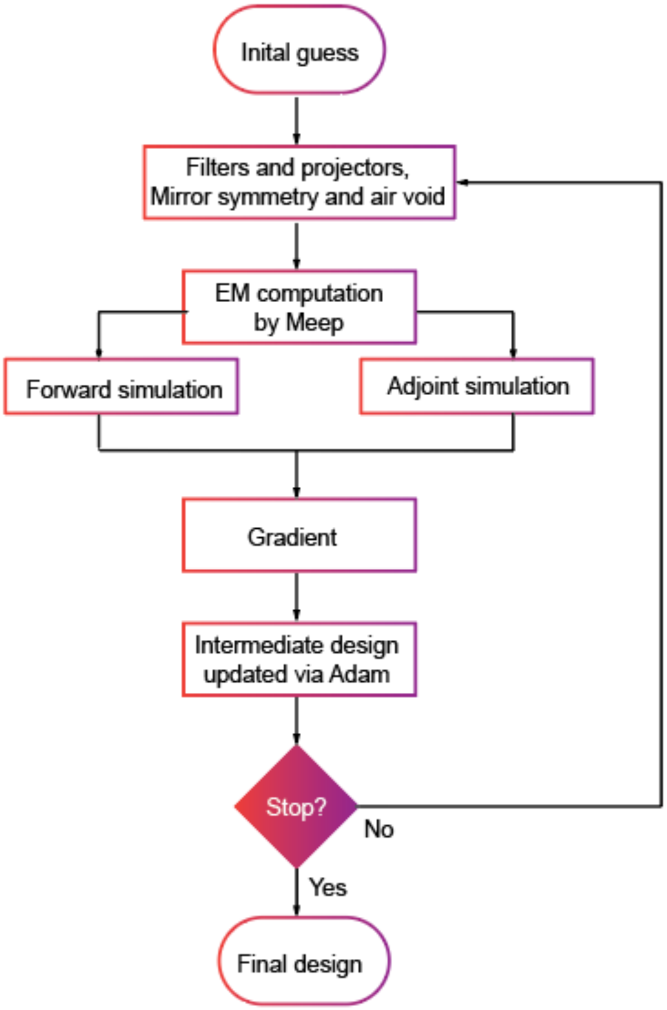

Figure S1| Optimization workflow.

Section 2: Chiral density enhancement spectrum

We plot the spectrum of the superchiral field in Fig. S2, which reveals a peak position (near 1140 nm) consistent with the wavelength at which the ED–MD phase condition (see Figure 2 in the main text) is satisfied.

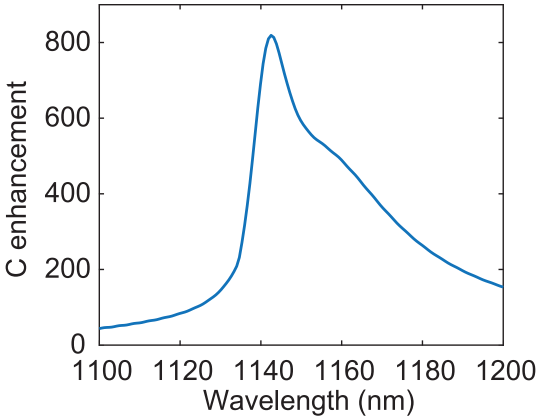

Figure S2| Chiral enhancement as a function of wavelength.

Section 3: Freeform element contribution analysis

We note that previous designs with a single element in each unit cell[1–3] exhibit modest chiral-field enhancements at the few-tens level. In our freeform design, the additional surrounding disks at the unit-cell corners play a critical role in further enhancing the chiral density. To validate this, we removed the surrounding disks to obtain an isolated holey disk at the center and analyzed its modal contents. As shown below in Fig. S3, the MD mode, which originally appeared as the dominant mode, disappears, leaving only a reduced ED contribution. In the absence of colinear magnetic component, the chiral enhancement drops to ~10. This confirms that the surrounding disks are essential for exciting the desired modes and for fine-tuning the relative ED–MD coupling dynamics.

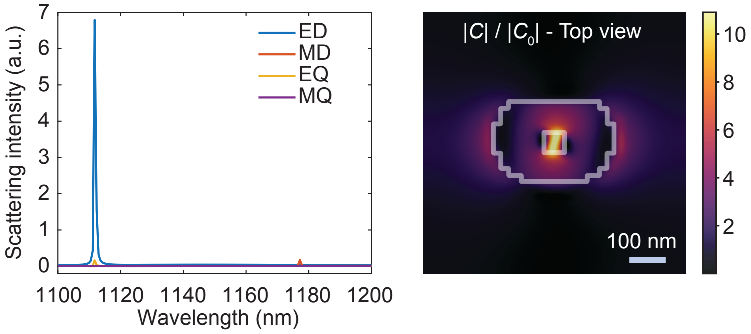

Figure S3| Multimodal analysis (left) and chiral density enhancement mapping (right, @ 1142 nm) for the design without the surrounding disks.

Section 4: Additional physically non-intuitive superchiral metasurface designs

To highlight the diverse freeform topologies accessible with our framework, we designed additional superchiral metasurfaces starting from different random initial conditions. As shown in Fig. S4 below, all designs exhibit more than 200× chiral density enhancement, although the hotspot magnitude and localization vary substantially, as is typical for non-convex optimization. Regarding the freeform topologies, some structures display sharper curvilinear features (edges and corners), while others exhibit smoother, more fabrication-friendly geometries. These examples demonstrate the significant diversity of locally optimal superchiral designs enabled by our freeform framework.

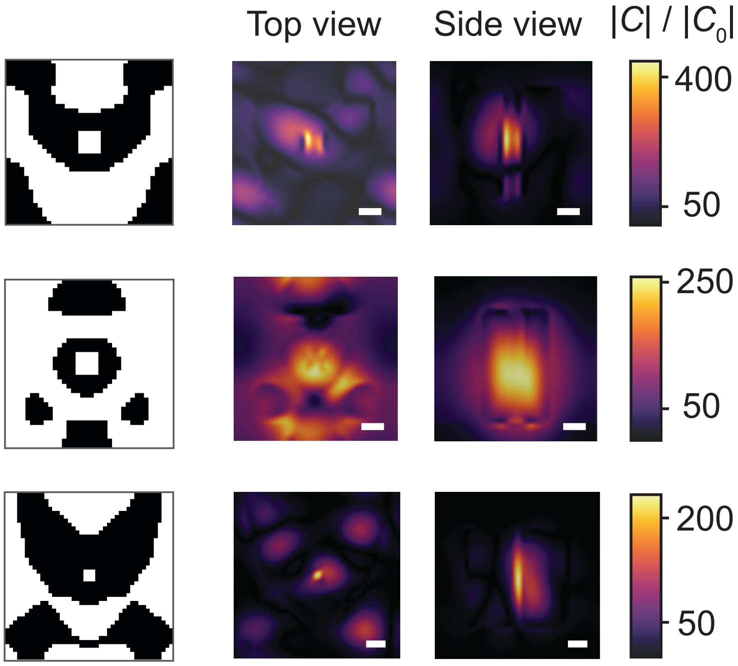

Figure S4| Additional freeform superchiral metasurface designs

Section 5: Experimental methods.

Fabrication: The freeform metasurfaces was fabricated on 462-nm thick amorphous silicon grown on a fused silica substrate using plasma-enhanced chemical vapor deposition. A 200-nm-thick PMMA 950K A4 was spin-coated at 4,500 r.p.m., followed by thermal evaporation of a 10-nm-thick chromium as a conduction layer. The nanostructure layout was defined by electron beam lithography, after which a 35-nm Al_2O_3 was evaporated by e-beam evaporation as an etch mask.

Silicon nanorods were subsequently etched by reactive ion etching using a mixed gas of SF_6 and C_4F_8.

Chiral analyte preparation: The chiral molecules (S)-(+)-1,2-Propanediol, (R)-(-)-1,2-Propanediol were obtained from Sigma-Aldrich (No:540250) and Thermo Scientific (No: AAB2193706), respectively. The molecules were diluted in dimethyl sulfoxide (Thermo Scientific, No: 032434.K2) to form a mixed chiral solution, which was then drop-casted onto the metasurfaces for sensing measurement.

Optical characterization: Schematic of the optical setup is shown in Fig. S5 below. The device is illuminated with CPL from a supercontinuum source (NKT SuperK EVO) and imaged through a microscopy system consisting of a 20× objective (Mitutoyo) and a tube lens. The transmitted signal is split by a beam splitter, and one arm is collected with a spectrometer.

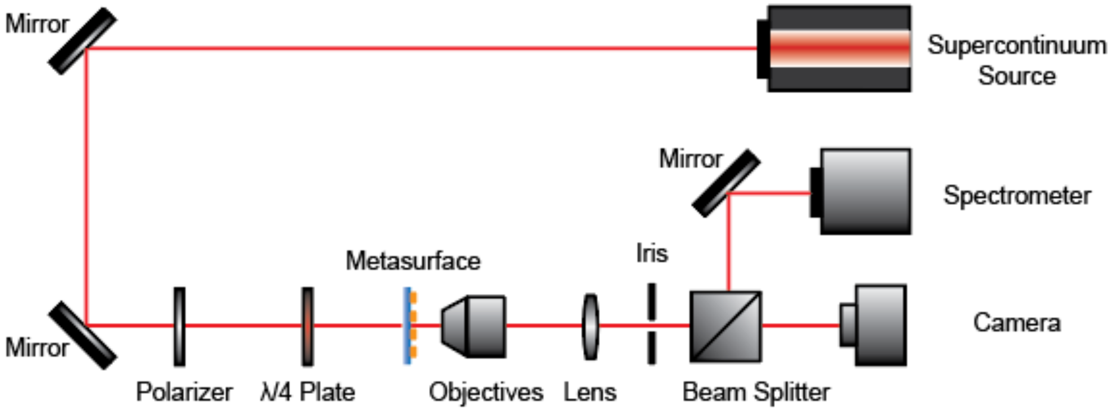

Figure S5| Optical set-up for dichroism spectroscopy. λ/4 plate: quarter wave plate.

Section 6: Chiral sensing performance of the initialized design

We analyze the chiral sensing performance of the initialized starting point used in the topology optimization. The initial design starts from a uniform index distribution with n=2.25 and a centered air void with n=1 (Fig. S6a). The chiral density (Fig. S6b and S6c) shows no obvious enhancement

compared to that of free-space CPL. The CD signals (Fig. S6d) for the molecules alone (orange lines) and for molecules coated on the initialized metasurface (blue lines) are comparable and both negligible (on the order of 10^{-5}), compared to >100-fold enhancement by the optimized metasurface. These results highlight the challenge to enhance chiral density through empirical structural design and underscore how our optimization framework effectively explores the freeform design space to push the chiral enhancement limit.

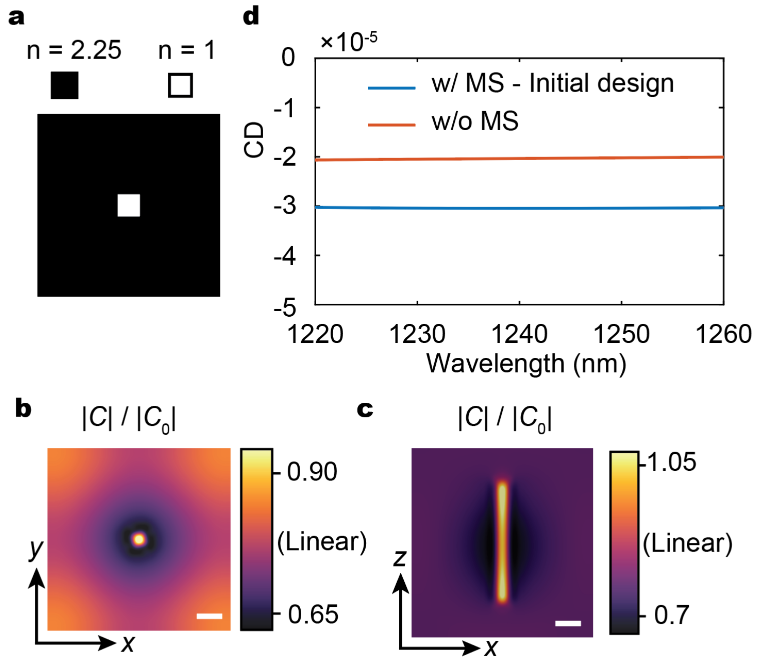

Figure S6| Sensing performance of the initial design. Scale bars: 100 nm.

Section 7: Chiral sensing with varying Pasteur parameter values

In our simulations, the adopted Pasteur parameter values were not experimentally measured; they were selected as an effective parameter to model a weakly chiral system. We focus on a small $\kappa \sim 10^{-3}$ to highlight the ultrasensitive capability of our sensor. We note that the exact Pasteur parameter values are not critical to our sensor performance improvement. As shown in Figure S7, strong CD signals can be obtained for a wide range of Pasteur parameters. The peak spectral positions remain constant with the κ values only affecting the contrast. The sensing signals with

these choices are all enhanced over two orders of magnitude compared to a bare un-patterned substrate. The specific parameter values $\kappa = (7 - 1.5i) \cdot 10^{-3}$ were then selected to align the CD contrast with our experiments shown in Figure 3h.

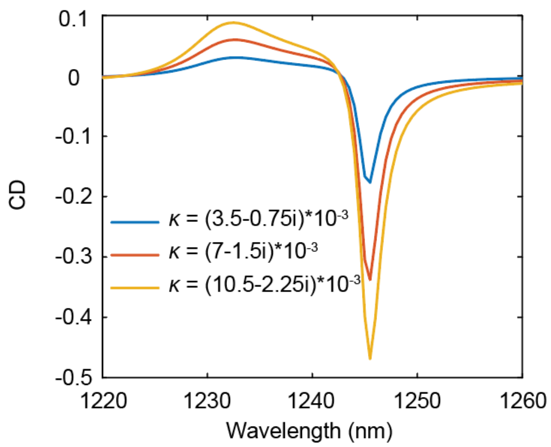

Figure. S7 | CD dependence on the Pasteur parameter κ.

Section 8: Chiral enhancement under geometric perturbations

We attribute the measured spectral shifts to fabrication imperfections. To validate the effects from non-ideal lithography, we evaluate the CD by changing the center gap sizes of the freeform structures. As shown in Fig. S8 below, we observe noticeable shifts (~10 nm) in the CD peak positions under a 10 nm size variation. We also note that the CD bandwidth becomes narrower for larger gaps. However, the required precision in gap size around the nominal 60 nm value is within our EBL capability. Similarly, other geometrical imperfections, such as feature dilation/erosion and surface roughness, will collectively shift the spectral peak.

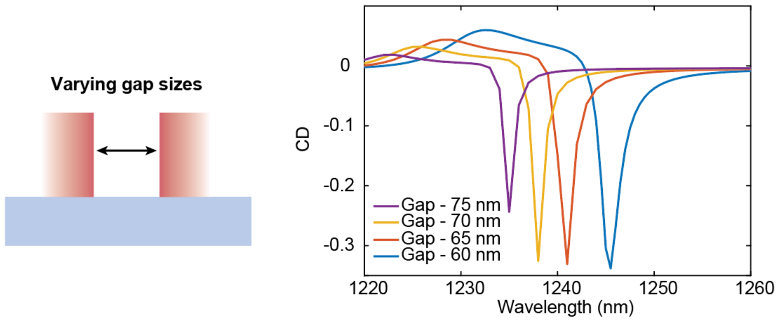

Figure S8| CD spectra versus air gap sizes

Section 9: Assessment of residual metasurface chirality

To validate that the measured circular dichroism (CD) arises solely from the chiral molecules, rather than residual device CD due to experimental imperfections, we recorded the CD spectra of the achiral metasurface without analyte and compared it with the analyte on bare glass and on the metasurface. As shown in Fig. S9, the metasurface-only control exhibits negligible CD within the measurement uncertainty, verifying that the observed CD stems mainly from the chiral analytes.

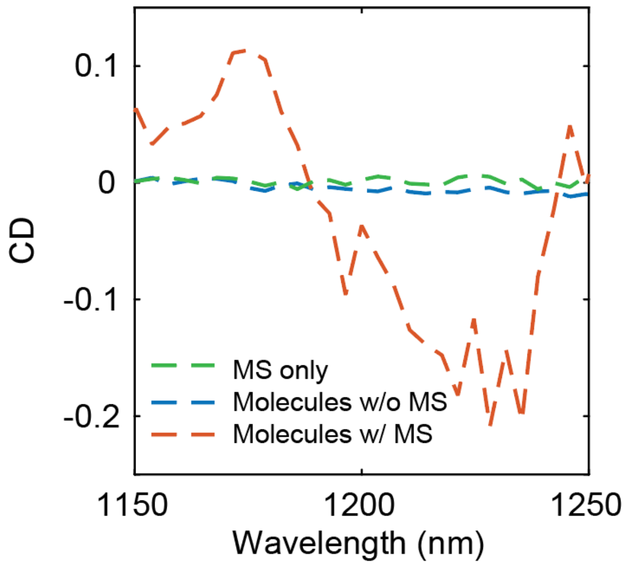

Figure S9| Measured CD spectra of the metasurface alone (green), molecules on bare glass (blue), and molecules on the metasurface (orange).

Section 10: Assessment of residual chiral molecules from the cleaning procedure

To ensure that our sensor is free of residual chiral adsorbates before each measurement, we tested several sample-cleaning strategies to establish a thorough rinsing protocol and converged on a procedure involving sequential use of dimethyl sulfoxide (DMSO), acetone, isopropyl alcohol (IPA), and deionized water. The optical spectra of a dummy device used for trial-and-error testing over multiple rinsing cycles are shown in Fig. S10. The spectra remain almost identical after each cleaning cycle, confirming a reliable rinsing procedure for repeated measurements.

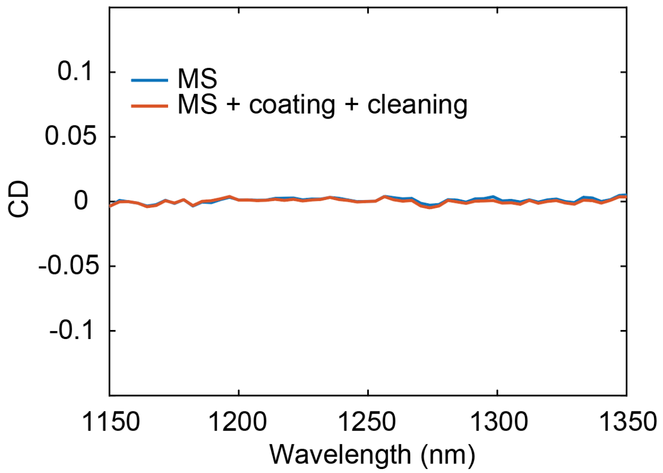

Figure S10. CD spectra before (blue line) and after (orange line) coating and cleaning procedures.

References

1. Mohammadi, E. *et al.* Accessible Superchiral Near-Fields Driven by Tailored Electric and Magnetic Resonances in All-Dielectric Nanostructures. *ACS Photonics* 6, 1939–1946 (2019).

2. Mohammadi, E. *et al.* Nanophotonic Platforms for Enhanced Chiral Sensing. *ACS Photonics* 5, 2669–2675 (2018).

3. Solomon, M. L., Hu, J., Lawrence, M., García-Etxarri, A. & Dionne, J. A. Enantiospecific optical enhancement of chiral sensing and separation with dielectric metasurfaces. *ACS Photonics* 6, 43–49 (2018).
